# Case of Paracentesis Thoracis

**Published:** 1853-09

**Authors:** John T. Metcalfe

**Affiliations:** Physician to Bellevue Hospital


*Case of Paracentesis Thoracis.*—By JOHN T. METCALFE, M. D., Physician to Bellevue Hospital.

CASE I.—*Aneurism of the Ascending Aorta, Serous Effusions in right pleura.* Thoracentesis for relief of Orthopnœa.

Patrick Kelley, æt. 50, tailor, a native of Ireland, presented himself at the clinique of the University Medical College, June 13, 1852, complaining of dyspnœa and cough.

The patient stated that his health had been good, with the exception of an attack of ague, fifteen years since, until about the beginning of February last. He was then taken with a dry, hacking cough, which was soon followed by œdema, especially of the lower extremities and right arm, and by dyspnœa. These symptoms had gradually increased, until the present time, and had lately, been accompanied by violent palpitation of the heart on taking a brisk walk, or on going up stairs. He had, also, found much difficulty in lying on his back, or on the left side; and had complained of lancinating pain about the right nipple, and of great œdema of the right side of the face.

Of late, the above symptoms had become aggravated, his nights had been restless, his appetite had failed, the bowels had been constipated, and the urine scanty and high colored.

At the time of making the examination, the pulse was of natural frequency, perfectly regular, equal in the two wrists, in size and strength. Respiration, 40 in the minute. Inspection showed that the chest was nearly symmetrical. The jugular veins (of the right side, especially) were much distended, but did not pulsate. Many tortuous veins of small size ramified over the right side of the chest and abdomen. There were unusual developments of the mammary and superficial epigastric vessels. The right side of the face and right arm were considerably anasarcous. On forced inspiration, the left side of the chest expanded more freely, and showed the intercostal depressions more plainly than the right.

Percussion was normally resonant over the left chest. Auscultation revealed nothing unnatural, except puerile respiration. On the right chest, anteriorily, as far down as the fifth rib, percussion heavy, respiratory murmur weak, voice natural. No rales.—Heart-sounds transmitted to the infra-clavicular region, with considerable distinctness.

Posteriorly, below the upper third of chest, percussion was dull. Over the same space, there existed bronchial respiration, broncophony, and clear transmission of the heart-sounds. By placing the patient on all fours, the last mentioned physical signs were changed for greatly diminished dullness on percussion; and by the almost complete disappearance of the voices and breath-sounds, noticed whilst in the erect posture. A very careful examination failed to detect any morbid vascular or cardiac phenomena, or any localized dulness by the pleximeter, other than have been already mentioned.

The diagnosis made before the class was, "Serous effusion into the right pleural sac, due to some abnormal growth at the root of the right lung, probably of a carcinomatous nature."

The patient was treated by a member of the clinical class, Mr. R. B. Conn, under my direction, with a view to effecting absorption of the contents of the pleura. Blisters, at first of considerable size—subsequently, small, flying ones, were applied to the chest. Hydragogue cathartics and diuretics (among which the salts of potash, digitalis, and achillea millefolium may be mentioned) were freely employed, their use having been preceded by gentle ptyalism. From this treatment, which lasted about three months, there was no benefit derived. On visiting the patient from time to time, I ascertained that the general symptoms remained very much as they were on his applying at the clinique.—There was gradually increasing dyspnœa, lately, amounting to orthopnœa; the effusion seemed to be increased in extent; there was more œdema of the right side of the face; and the right arm and breast had become more anasarcous. It was also observed that the heart-sounds were more and more distinctly audible over the middle of the right third costal cartilage, and that there was slight dullness on percussion, with a slightly perceptible impulse over the same region.

The condition of the patient not having been improved, but, on the contrary, an aggravation of all the prominent symptoms having taken place, I determined to see if relief would not follow the withdrawal of what fluid might be found in the pleural sac. This was accordingly decided on, and at the suggestion of my friend Dr. J. Edward Weber, I concluded to use the trochar of Schuh, with the substitution of a flexible catheter for the trough of the Vienna Professor.

The following are notes of the physical examination made on the morning of the operation (August 14th). Present, Drs. Weber, James R. Wood, and Van Buren. Pulse 110, small, equal in two wrists. Respiration 40. Considerable œdema of the right arm, which measured two inches more in circumference than the left at the elbow. Right side of the face and neck œdematous. Jugular veins greatly distended, especially on the side last mentioned, where there is much lividity of the skin. The epigastric and mammary veins much enlarged. No motion of respiratory expansion on right side, where the clavicle was scarcely visible, and the general surface smooth; contrasting, in this, with the opposite anterior half of the thorax, where the natural conformation and much depression of the intercostals spaces were seen during respiration. One inch below nipples, circumference of affected side two inches greater than that of its opposite. In right chest, percussion abnormally clear over infra-clavicular region, as far as upper border of third rib—below this, flat. Over resonant portion, vocal fremitus marked, no palpitation; elsewhere, absent. At the same part, vascular murmur weak; over lower three-fourths of lung, anteriorly and posteriorly, dull percussion, bronchial respiration, and broncophony ,extending nearly to the diaphragm. Transmission of heart-sounds, without murmur (this has never yet been detected) very plain, at the right nipple, which is three lines further from the median line of the body than the left. The left side of the thorax gives normal results on percussion and auscultation, with the exception of general puerile respiration.

The patient having been seated sideways on a chair, was directed to make a full inspiration, and to hold the breath at the end of it, in order that no involuntary inspiratory effort might cause admission of air into the pleural cavity. The trochar was introduced by a firm plunge, between the sixth and seventh ribs, just under the angle of scapula. At first I miscalculated the depth necessary to introduce the instrument, in order to reach the fluid, not having caused it to penetrate more than an inch. On account of the ædematous state of the thoracic parietes, it was found requisite to insert one and three-quarter inches of the trochar. This was withdrawn, the stop-cock turned, and a flexible catheter, having its lower end submerged in a little warm water, was attached to the outer extremity of the canula. On opening the tube, by turning the cock, a few bubbles of air contained in the catheter escaped from the lower end, and were followed by a stream of pale straw-colored serum.

The patient complained of a good deal of pain when the puncture was made, and was troubled for a few minutes with a short, dry cough. The fluid was permitted to flow until percussion showed that it had reached the level of the puncture in the chest. A glass of brandy and water was given him, the instrument withdrawn, the orifice covered by adhesive plaster, over which was put a compress and roller bandage, after which Kelly expressed himself as feeling very comfortably. The pulse was then 106, respiration 40.

The fluid evacuated, amounted, by careful measurement, to *forty-eight ounces*, and was nearly entirely solidified when heated in a spoon.

The kidneys had not acted well for the first week; and at Dr. Weber's suggestion, the following prescription was administered as a diuretic:

℞ Ammoniæ Sesquicarb. ʒ i.

Aquæ Communis, ℥ vi.

M.

S. A tablespoonful, every two hours.

9, P. M. Had gone to bed after the operation. Was somewhat distressed from an uneasy, but not painful feeling in the chest —coughed a little—ordered him a tumbler of milk-gin punch, and a quarter of a grain of súlphate of morphia.

15th. 10, A. M. Has passed a quiet, comfortable night, and has been able to lie down at full length and to sleep, from about 10 o'clock the evening before. Expresses great satisfaction at relief from the distressing orthopnæa, under which he he has so long labored. The secretion of the urine much more copious than it has been for some time.

The administration of the carbonate of ammonia and gin-punch was continued for about a week, at the end of which time, the œdema had nearly all subsided from the face, chest and right arm. Sleep had been nearly natural, and the prospect of being soon able to resume his work seemed fair. It is now observed, that there was an impulse communicated to the hand, when applied over the sternal border of the right infra-clavicular region, where a slight local prominence was observable, on inspection. At this part for about an inch and a half square, there was a slight dullness on percussion. No abnormal murmurs recognized. Distention of jugular veins still very considerable.

The aneurismal nature of the tumor was now pretty obvious.— From this time, the patient's condition remained tolerably comfortable until the early part of September, when he contracted a severe dysentery. This was found to be very little amenable to treatment, and finally terminated his life, apparently by exhaustion, on the 9th of October, 1852.

After the operation, there was never any very great complaint of dyspnœa. The patient was able to sleep in the recumbent posture; and until prevented by debility, consequent on the dysentery, had no difficulty in walking about his room. There was no change in the physical signs mentioned at the time of the operation, except the diminution of dullness on percussion, to a slight degree. The bronchial respiration and broncophony remained as evident as the had ever been. Trifling increase in the tumor, thought to be aneurismal, over the spot where the impulse was noted—the heart-sounds (?) were, towards the last, very distinct.

*Sectio Cadaveris*—11 hours post mortem.

Body pale; no œdema of arm or chest; no venous distention of small vessels of chest or abdomen.

*Head*—Not examined.

*Thorax.*—Lungs adherent to costal walls by old false membranes of great strength, on the left side the adhesion universal.—On the right side the lung was adherent to the thoracic parietes from the summit to the upper border of the fifth rib. Below this, there was a cavity formed by the base of the lung, superiorly; the upper surface of the diaphragm, inferiorly, and by the costal pleura laterally. This cavity contained ℥xxij of clear transparent serum, similar, in every respect to that removed at the thoracentesis.—The heart, in situ, was smaller than the average size, (℥vi. in weight) and presented clearly marked fatty degeneration of the muscular fibres under the microscope.

Commencing one inch and a half from the valves of the aorta, was an aneurism of that vessel, springing from the right side, spreading posteriorly and anteriorly. It was filled with loosely packed layers of fibrine, and was as large as a goose's egg. Opposite the right costal cartilage, adhesion between the sac and wall of the chest had taken place over a place as large as a Spanish dollar, where the wall of the artery had been absorbed. Beyond the aneurismal sac, there was enlargement, with athorma and calcification of the aorta, as far as it could be traced, in the thoracic portion.

The position and size of the tumor had been such as to cause very trifling pressure on the trachea or œsophagus; but the superior cava, the right bronchus, and the pulmonary veins of the right side, were much compressed.

The operation in this case was simply intended to have a palliative effect. It was considered up to the time of its performance that the patient was laboring under a disease of incurable nature; and it was thought evident that much of the distressing difficulties of breathing was due to the effusion in the chest. We may here make the same remark that Dr. Henry I. Bowditch's observation has suggested—that it is not necessary for the relief of dyspnœa, due to this cause, that the fluid should not re-accumulate. The relief given by its first withdrawal is much more permanent than we should *a priori* be led to expect, in cases like Kelly's, where the liquid agains returns in the pleural sac.

It will be observed that the phenomenon of abnormally *increased resonance*, on percussion, in the infra-clavicular region of the diseased side existed in this case. The explanation of this sign, as regards its physical cause, is as yet, not very satisfactory. Its existence has long been known, and an interesting paper touching this point, has lately been published by Dr. Markham, in the Monthly Journal of Medical Science. In this case it certainly was due to the effusion or secretion of air into the pleural cavity, as the costal and pulmonary surfaces were closely adherent by old organized fibrin.

It was not unlikely that this unnatural clearness of the percussion note was the cause of my failing to recognize, at an earlier period, the presence of the aneurismal tumor.

Case II.—James Somerindyke, native New York, a gardner by occupation, entered Bellevue Hospital on the 27th of January, 1853, suffering from pain and soreness in the præcordial region, with dyspnœa.

With the exception of an occasional attack of rheumatism or cold such as his out-door life exposed him to, he had enjoyed good health, until twelve days before admission; when he had been taken with a violent fit of vomiting, during which he strained very considerably. After this, he observed that the left cheek, anteriorly, was sore and painful. He referred the attack of vomiting to having suddenly encountered a very disagreeable odor.

One week subsequently, whilst driving a horse and cart, having his hand on the latter and making very moderate pressure in pushing, he suddenly found himself suffering from dyspnœa, but without pain—to use his words, "I felt just as if I had run a hard foot race." He sat down to rest, for about a quarter of an hour, without obtaining relief from the difficulty of breathing, which became so considerable, that he was obliged to drive home in his cart. Repose gave no respite from this trouble, and he concluded to enter the hospital, under the care of Dr. Clark.

On admission, he complained of inability to lie down, the attempt being followed by a feeling of threatened suffocation. The slightest muscular exertion increases greatly the difficulty of respiration, and præcordial pain and distress. Orthopnœa constant; pulse 160, small, irregular, intermitting. The treatment ordered was absolute rest, dry cups to the præcordial region, and the internal use of extract of hyoscyamus.

March 18th.—Since last note, the patient's general condition has somewhat improved. He is now able to sleep in a semi-recumbent posture. From this, however, he is often suddenly aroused, with a start, and has a violent dry cough and dyspnœa. These symptoms are relieved by sitting or standing upright. Physical examination by Dr. Clark:

Position of apex-beat difficult to localize palpitation. It is about four inches from the medium line of the body, to the left. Systolic impulse increased in force at the apex, and at no other point. By ordinary percussion, vertical diameter of heart's dullness 4½ inches—transverse not exactly determinable. Auscultation gives no endocardial murmur, nor any other abnormal sound. Entire loss of rhythm and extremest irregularity of heart's action. Former treatment (palliative) pursued. The disease was considered to be one affecting the valvular apparatus; probably rupture.

By the beginning of May, the patient had been considerably relieved; so much so, that he determined to leave the hospital, and to return to his work. This he was able to attend to, but very imperfectly. A few days after discharge, whilst making some slight exertion, he was again taken with the præcordial pain, dyspnœa and cough. He had several attacks of epistaxis, his distress continued to increase, and on the 16th of May, he entered as a patient in my service. The following notes was then made: body somewhat emaciated; face anxious in expression, with slight lividity; arcus senilis well marked in both eyes; feet œdematous; dyspnœa great; heart's impulse diffused, but not strong; apex beats two inches to the left of its natural position; præcordial dullness as before observed. Elsewhere, chest resonant anteriorly, except over the upper sternal region, where dullness on percussion is marked. Posteriorly there is resonance, except in the right infrascapular region. Respiratory mumur puerile, save where dull percussion has been noted; in these portions of the chest it is absent. In the interscapular spaces (especially in the right,) prolonged expiration blowing in character, with occasional sonorous rhonchus. Heart-sounds (?) transmitted with unusual distinctness to middle of right clavicle, and generally to the right side of the chest. The second sound, over the position of the valves, not clearly recognizable. Rhythm and character, as when examined by Dr. Clark.—No abnormal murmur. The signs lead to the opinion that there was effusion in the right pleura and dilatation of the arch of the aorta.

The patient was treated by the administaation of Hoffman's Anodyne, in drachm doses, to relieve the sudden paroxysmal fits of dyspnœa under which he labored. This medicine, after a short time, had to be discontinued, on account of the unpleasant cerebral excitement and wildness it produced. In its place the solution of sulphate of morphia was prescribed; generally with temporary relief. To combat the tendency to dropsy, hydragogue cathartics were administered occasionally, whilst, as a habit, he took the infusion of digitalis, in half ounce doses, three times a day. Freer watery evacuations were obtained from the former, and copious diuresis by the latter means. Nevertheless the legs began to swell, becoming very greatly distended and hard, the genitals became anasarcous, there was ascites and evident increase in the fluid effusion in the right pleura, the orthopnœa was constant and painful to behold.

On the 6th of July at my visit, I found an aggravation of all Somerindyke's symptoms. The extremities were cold and livid. The most violent action of all the muscles of respiration was necessary to enable him to breath at all. The dropsy, generally, increased since last observation. On physical examination there was found to be a great increase of fluid in the right pleural sac. The right chest measured, on a level with the fourth rib, three quarters of an inch more than the left. *It expanded, on forced inspiration, as much as the opposite side, and showed the intercostal depressions quite as plainly, without widening of the spaces between the ribs.* Strong visible and palpable pulsation of the carotid and subclavian arterteries of each side, especially of the right. These full, hard, powerfully-beating vessels contrasted strongly with the small, irregular and weak radial pulse, which latter is similar, in every respect, in the two wrists.

The left side of the chest furnished natural signs on palpation and percussion, with puerile respiration over its whole extent. The right side was dull from the level of the third rib, to the abdomen. Over this portion there was soft, distant, bronchial breathing and broncophony. In the axillary region, faint œgophony. No vibration of chest wall, on palpation over lower three-fourths of right side. Respiratory murmur weak over resonant part of right lung.

Heart's dullness as before noted. Over the whole præcordial space, a faint systolic murmur, transmitted to subclavian and carotid arteries, and more audible, perhaps, over third costo-sternal articulation than elsewhere. Second sound nowhere recognized, no thrill in palpation. Impulse weak. Pulse 84, respiration 40. The patient's condition was evidently such that no benefit was to be expected from the use of the hydragogues or diuretics. He was anxious to have something done to afford relief from the feeling of suffocation; and caught eagerly at the idea of having an operation performed, which promised any chance of rendering him more comfortable.

According, having placed him on a stool of convenient height,

I introduced an exploring needle between the seventh and eight ribs, immediately beneath the angle of the scapula. Along the groove of this a drop of clear serous fluid escaped. I then had a bandage applied around the chest, and into the puncture made by the needle, I plunged the trochur of Schuh, to the depth of one inch, having previously, with a lancet, made a short incision through the skin. The introduction of the instrument appeared to give great pain, and caused such movements of the body as to make it necessary to have him held by assistants. On turning the stop-cock, I experienced the same difficulty as had been met with in Kelly's case. Either the instrument had not penetrated to a sufficient depth to reach the fluid, or the motions of the patient had caused its withdrawal. I replaced the trochar in the canula, and pushed it half an inch deeper. On its withdrawal and turning the cock, a full stream of limpid serum flowed from the canula. The catheter was now adjusted, its free end immersed in a little clean water, and the fluid allowed to escape until the chest was emptied. In this manner *seventy-two ounces* of serum were evacuated. During its flow, steady tension of the roller bandage was kept up. The patient, I think, was by this expedient relieved from the sense of discomfort in the thorax, which, in my former case, and in several of Dr. Bowditch's,* was so marked a symptom.

At the close of the operation, which lasted half an hour, the respirations per minute had fallen to 26, and nearly all trace of dyspnœa had subsided. Great relief was visible and was expressed at being able to take a long, full inspiration.

The chest, examined in few minutes after the operation, showed the percussion sound to be symmetrical on the two sides. Vesicular murmur pure, but faint, over whole of right lung. Vocal resonance normal. No bronchial souffle. 5½ o'clock, P. M. Patient was seized with a severe chill, which lasted about twenty minutes, and was accompanied by occasional sharp, darting pains in the neighborhood of the puncture. In the course of the evening, the skin became hot, except that of the extremities, which remained cool. Pulse 100, respiration 30. The bandage was felt to be too tight around the chest, causing by its pressure a certain amount of dyspnœa. In loosening it, relief was afforded. To take twenty drops of solution of morphia. (Grs. xvi. to ℥ i.)

July 7th. Has rested comfortably during the night, in the horizontal posture, and has not suffered from dyspnœa. Some sharp pains of a lancinating character, extending from the right iliac to left hypochondriac regions. Has slight pain about right nipple, which was relieved by loosening the bandage still more.

* American Journal of Medical Sciences, April, 1852.

Pulse 96, respiration 24. To repeat the morphine at bed-time. To relieve the anasarca of the abdomen and inferior extremities, a half dozen small lancet punctures were made in the skin of the calf of each leg. Serum dropped freely from these.

July 8. He passed a perfectly comfortable night in sound sleep. No pain felt, even on forced inspiration.

July 18. Improvement continues. For several nights has not needed the anodyne. Large quantities of serum have flowed from the punctures without producing erysipelas. Abdomen and thighs much swollen and softer.

In this case also, the operative procedure was simply intended to serve as a palliative. This it has certainly done in a most marked manner, and after signal failure of those means on which we commonly rely in analogous cases.

I do not remember to have met with another instance, in which any thing like so considerable an amount of effusion into the pleural sac has been found to exist, without the presence of the signs furnished by inspection, noted as being absent in Somerindyke's case. Surely, with more than two quarts of serum, the accumulation dating from nearly two months previously, we should have been led to expect widening of the space and inaction of the intercostal muscles, displacement of the liver and mediastinum, and more considerable increase in the semi-circumference of the chest, on the affected side.

I have spoken, incidentally, of the instrument, used in these operations. It is composed of a trochar, such as is ordinary employed for tapping in hydrocele, a little over one line in diameter, and three and a half inches long. This fits into a canula, to which is adapted a stop-cock, at the distance of two inches and a quarter from the inserted end. Between this point and the other extremity, a roughened handle projects at right angles to the axis of the tube. There is no cup, as in ordinary canulæ, to this end; but the ordinary diameter is preserved, so as to allow the adaptation, over it, of the elastic catheter. This has been preferred to Schuh's trough, as being more simple, more easily obtained, and less likely to get out of order.

It will readily be imagined that the object of the procedure mentioned is to remove the fluid from the chest, without allowing the entrance of air and incurring the risk of making a pyogenic membrance of the pleura. This end is easily obtained in the manner described, as it has been already effected by the plans of Raciborski, of Schuh, and of Bowditch. I conceive that its merit consists in its greater simplicity.

With regard to the exploratory puncture, I may state that it was made not from any doubt as to the existence of fluid, but that I might not introduce the trochar at a point where costal and pulmonary pleura should be adherent, one to the other, and thus oblige me to make a second use of the instrument. This has always given considerable pain—the needle is not complained of at all. It is not always easy to tell when the fluid has ceased to flow, unless by raising the free end of the catheter, and thus incurring the risk of allowing air to enter the pleural cavity. I have found that this can be done by rolling a piece of bread crumb between the fingers, until it is made slightly heavier than water, and letting this fall to the bottom of the fluid. It is only necessary to bring the end of the tube near the crumb, in order to detect the exit of fluid, by the motion imparted to the little mass.

It will probably be found that the use of the bandage, to compress the chest during the flow of the fluid, will be as beneficial, in this case, as it is now recognized to be, in paracentesis abdominis.

It was my intention to publish in full, with the preceding cases, two others, in which I made use of the same instrument, for almost the same disease. An unfortunate loss of my notes prevents me from giving more than an outline.

Case III.—In one, that of a young man suffering under enormous distention of the right side, by pleuritic effusion, producing orthopnœa, and threatening suffocation, I removed between forty and fifty ounces of yellowish, somewhat turbid serum. For a week afterwards, he was very comfortable, and slept with ease, in the recumbent posture. By imprudent exposure to cold and wet, he contracted peritonitis at the end of that time, and died after three or four day's illness. About half the amount of fluid removed had re-accumulated.

Case IV.—The second case was that of a child, three years old with accumulation in the left pleura so considerable, as to dislocate the heart's apex to the right nipple, to cause a descent of the liver below the costal margin, and to paralyze the intercostal muscles of the diseased side. The fluid removed from him was very much like pea soup in color and consistence. It amounted to forty-six ounces, and was slightly fetid. The respiration with him amounted to sixty per minute, and so great was the dyspnœa that the child was obliged to remain almost constantly in his cradle. In this case the operation gave such relief, that, in three days, he was able to walk out and spend the day with some children of his own age, in playing about their nursery. Owing to his struggles, the catheter was at one time displaced, and some twenty or thirty bubbles of air entered the pleural sac. By the expiratory movement they were soon expelled. No evil result followed this accident; nor do I believe that harm ensues from the entrance of air in cases like this, of true empyema.

In the course of a month, the fluid returned, in the case last mentioned. The parents of the child changed their habitation, and and I was unable to trace its history subsequently.

